# Note on Broncho-Pulmonary Spirochetosis (Bleeding Bronchitis)

**Published:** 1918-11

**Authors:** 


**Note on Broncho-Pulmonary Spirochetosis (Bleeding Bronchitis).** By H. Violle. *Bulletin de l'Académie de Médecine,* June 4th, 1918.

About ten years ago Castellani discovered in India a form of bronchitis due to a new microbe " the spirochaeta bronchialis ". In the first three months of 1918 the writer came across such cases at the naval hospital at Toulon, where more than thirty patients were suffering from this peculiar affection.

The most interesting feature of the disease is the presence of blood in the sputum. This sputum has an evenly pinkish tint, is viscous, and presents more or less the appearance of currant juice. In no other affection do we find so characteristic a coloration, hence its name of " bleeding bronchitis ".

It will be easily understood what mistakes are possible in the diagnosis of this form of bronchitis, as it recalls tuberculosis in its first stages. Half of our patients entered the hospital with a diagnosis of phthisis, suspected bronchitis or hemophthisis, and yet, after examination, no bacilli of tuberculosis could be traced in the sputa in any of the cases. On the other hand, an enormous quantity of spirochaetes of all forms and dimensions could be detected in the " frottis ", with nitrate of silver.

These organisms may be found on their own or mixed with other germs or desquamation cells, but they never affect the appearance or growth of fungi. They come with the disease and disappear with it. They are not to be traced in the nasal mucus, the urines, or the blood. The examination of the blood reveals only a slight anemia, probably due to the repeated hemoptisies.

This affection is of a fairly mild character; there is no temperature, and the general condition remains almost good ; but coughing at night is frequent, raucous, and rather painful; expectorations vary; they can be regular bleeding sputum, or muco-purulent, or muco-bleeding sputum.

The evolution takes about a month ; relapses are not unusual, and contagion is undeniable. Brought into France by Asiatic troops, it developed also among white men. One fourth of the cases were among French soldiers. Infection is propagated by a kind of spores, " coccoid bodies " of Laveran and Mesnil.

The gravity of the disease lies in the fact that all the open bleeding lesions made in the pulmonary tissues afford an ideal condition to receive germs of tuberculosis, pneumonia and other equally grave diseases.

